# BCN20000: Dermoscopic Lesions in the Wild

**DOI:** 10.1038/s41597-024-03387-w

**Published:** 2024-06-17

**Authors:** Carlos Hernández-Pérez, Marc Combalia, Sebastian Podlipnik, Noel C. F. Codella, Veronica Rotemberg, Allan C. Halpern, Ofer Reiter, Cristina Carrera, Alicia Barreiro, Brian Helba, Susana Puig, Veronica Vilaplana, Josep Malvehy

**Affiliations:** 1 Signal Theory and Communications, Universitat Politècnica de Catalunya, Barcelona, Spain; 2 Melanoma Unit, Dermatology Department, Hospital Clinic Barcelona, Universitat de Barcelona, IDIBAPS, Barcelona, Spain; 3 IBM Research AI, T Watson Research Center, Yorktown Heights, NY USA; 4 Dermatology Service, Department of Medicine, Memorial Sloan Kettering Cancer Center, New York, NY USA; 5 Kitware, Clifton Park, USA

**Keywords:** Melanoma, Cancer

## Abstract

Advancements in dermatological artificial intelligence research require high-quality and comprehensive datasets that mirror real-world clinical scenarios. We introduce a collection of 18,946 dermoscopic images spanning from 2010 to 2016, collated at the Hospital Clínic in Barcelona, Spain. The BCN20000 dataset aims to address the problem of unconstrained classification of dermoscopic images of skin cancer, including lesions in hard-to-diagnose locations such as those found in nails and mucosa, large lesions which do not fit in the aperture of the dermoscopy device, and hypo-pigmented lesions. Our dataset covers eight key diagnostic categories in dermoscopy, providing a diverse range of lesions for artificial intelligence model training. Furthermore, a ninth out-of-distribution (OOD) class is also present on the test set, comprised of lesions which could not be distinctively classified as any of the others. By providing a comprehensive collection of varied images, BCN20000 helps bridge the gap between the training data for machine learning models and the day-to-day practice of medical practitioners. Additionally, we present a set of baseline classifiers based on state-of-the-art neural networks, which can be extended by other researchers for further experimentation.

## Background & Summary

Skin cancer is one of the most frequent types of cancer and manifests mainly in areas of the skin most exposed to the sun. Despite not being the most frequent of the skin cancers, melanoma is responsible for 75% of deaths from skin tumours^1^. It can appear at any age, and it is the first most diagnosed cancer among patients in the 25–29 age group, the second among 20–24 years old, and the third among 15–19 years old^2^. Melanoma leads to significant years of productive life lost and stands as the costliest skin cancer in Europe in terms of cost per death^3^.

Given that skin cancer lesions are visible in the skin, dermatoscopy is a crucial tool in the diagnosis of skin cancer. This technique employs a magnifying lens and polarized light, penetrating deeper within the skin layers and minimizing surface reflection. Prior research has found that this technique allows enhanced visualization of the lesion structures, improving dermatologists’ diagnostic accuracy^4,5^. While experts typically look for specific structural and colour cues, standardized clinical procedures, such as the “ABCD rule of dermoscopy”, have been instrumental in skin cancer diagnosis^6^.

With the increasing use of high-resolution cameras and specialized dermoscopic adapters in medical institutions, there has been a surge in dermoscopic image data, prompting the development of computer vision algorithms for automatic lesion diagnosis^7–9^.

Earlier systems relied on the extraction of handcrafted features from skin lesions, similar to the rule sets that clinicians were using to perform diagnosis^10–13^. The aim was to develop specialized algorithms extracting colour, border features, symmetry, and other diagnostic criteria as inputs for machine learning classifiers. However, the growing availability of dermoscopic images has led to more sophisticated deep learning algorithms, particularly convolutional neural networks, that process images directly without relying on predefined rule sets. A significant catalyst for these algorithms’ adoption has been The International Skin Imaging Collaboration (ISIC), organizing annual challenges since 2016 for developing computer vision algorithms to classify and segment dermoscopic images of skin lesions^14–18^. Tschandl *et al*. showed that the performance of expert dermatologists confronted with dermoscopic images was outperformed by the top-scoring machine learning classifiers of the ISIC 2018 Challenge^16,19^. However, as highlighted by the authors, these algorithms underperformed on out-of-distribution images not represented in the training dataset of HAM10000^20^. A meta-study^21^ on the ISIC 2019 challenge^17^ has shown that state-of-the-art classification methods decrease by more than 20% on datasets specifically designed to better reflect clinical realities, as compared with a previous, well-controlled benchmark.

**Fig. 1:**
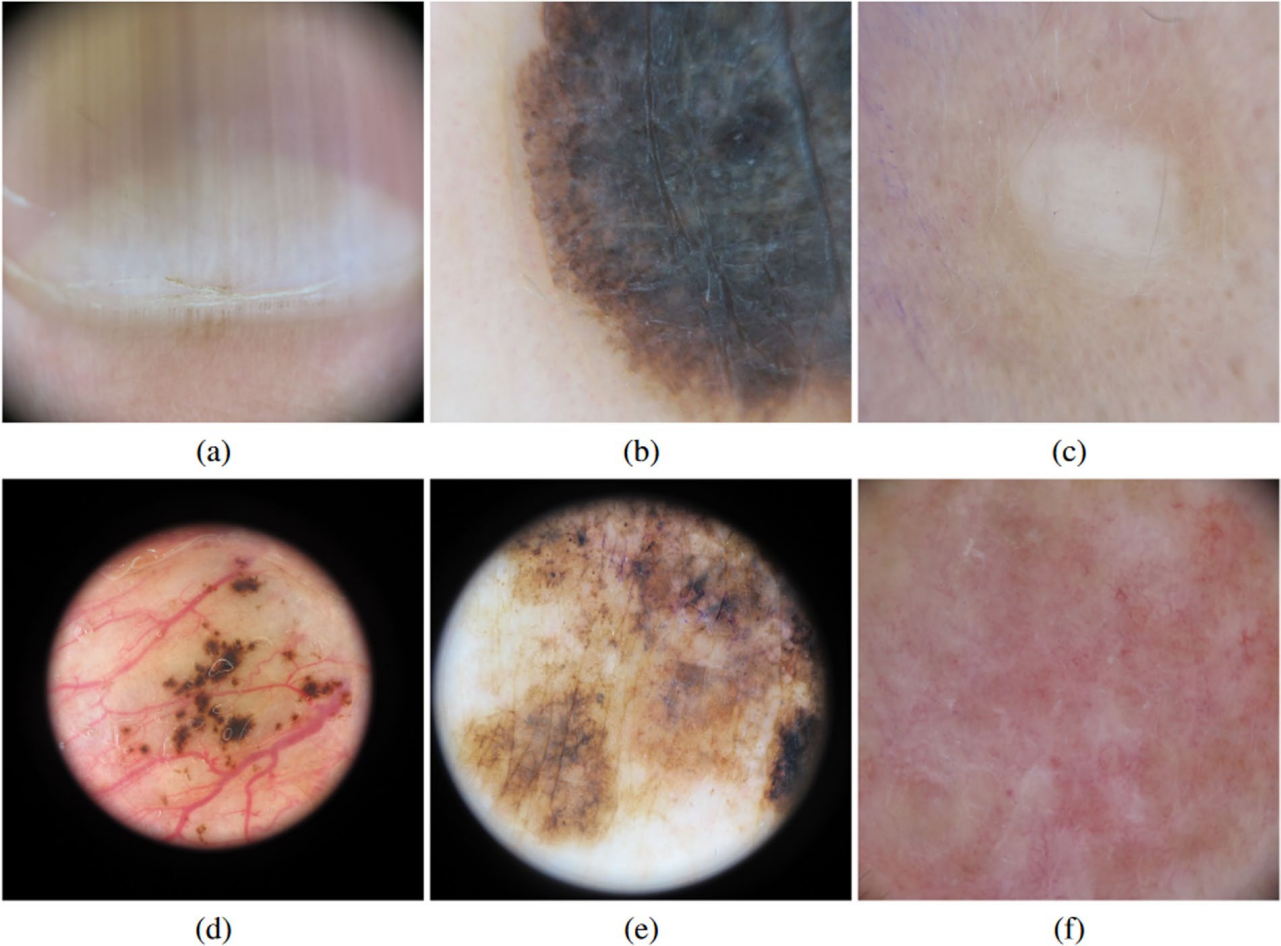
Dermoscopic images showcasing diagnostic challenges in BCN20000: (**a**) Lesion on a nail, (**d**) Lesion on mucosal tissue. (**b**) and (**e**) Lesions too extensive for the dermoscopy device aperture, illustrating size-related diagnostic obstacles. (**c**) and (**f**) present hypopigmented lesions.

The Hospital Clinic of Barcelona is a tertiary referral center. Its department of dermatology is responsible for treating high-risk melanoma patients. The hospital often receives challenging cases from other regional centers, making it a representative sample of lesions encountered across the region. The BCN20000 dataset addresses the challenge of unconstrained classification of dermoscopic images of skin cancer, capturing a wide array of lesions in diagnostically challenging locations (nails and mucosa), alongside not segmentable and hypopigmented lesions. Figure 1 showcases some of these diagnostic challenges. Most importantly, this dataset, aptly termed ‘dermoscopic lesions in the wild’, contains OOD images which occur in normal clinical practice but are not represented in current dermoscopic datasets.

**Fig. 2:**
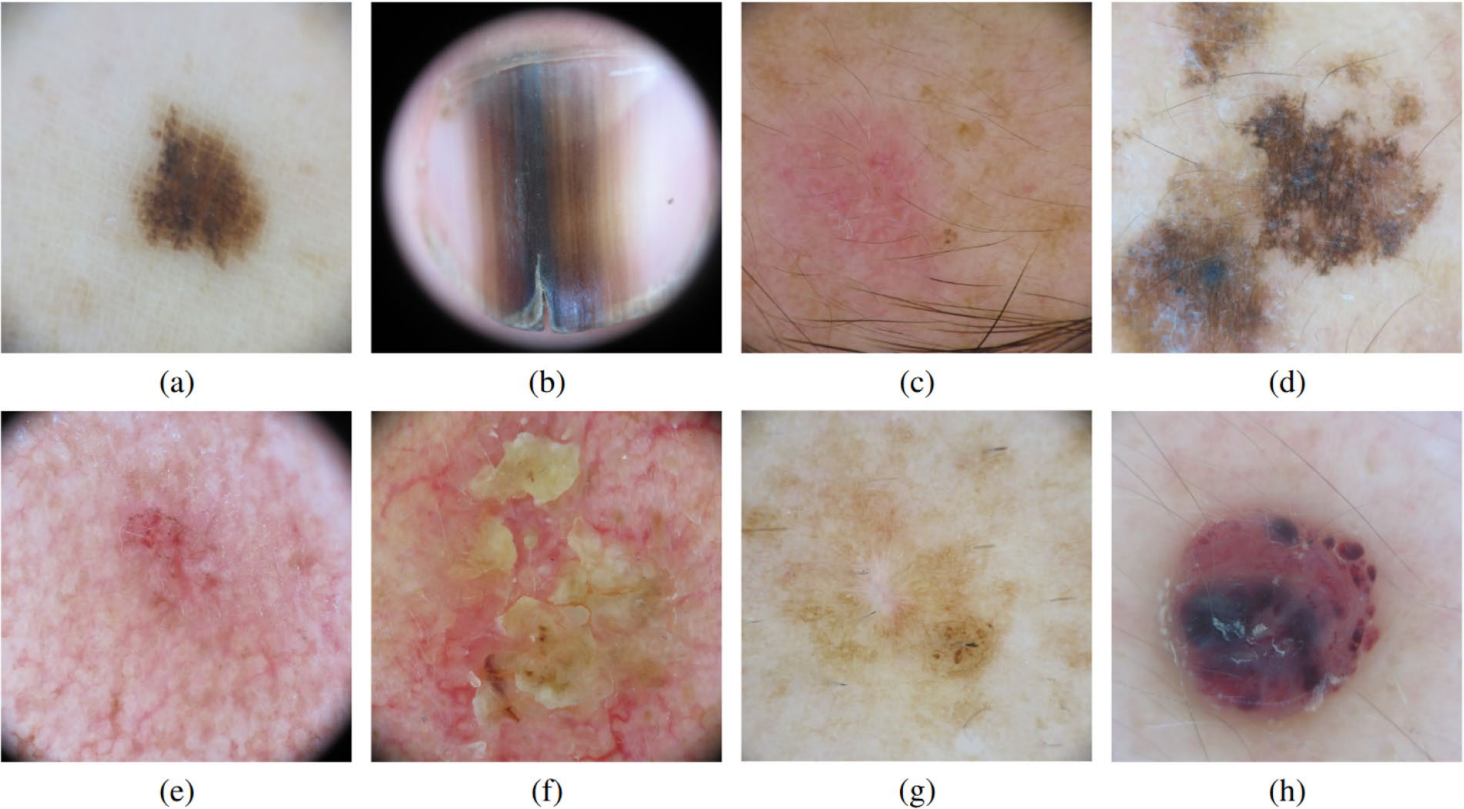
Samples from the BCN20000 dataset corresponding to (**a**) nevus, (**b**) melanoma, (**c**) basal cell carcinoma, (**d**) solar lentigo/ seborrheic keratosis, (**e**) actinic keratosis, (**f**) squamous cell carcinoma, (**g**) dermatofibroma and (**h**) vascular lesion.

Comprising 18946 dermoscopic images corresponding to 5583 skin lesions, the dataset includes nevus, melanoma, basal cell carcinoma, seborrheic keratosis, actinic keratosis, squamous cell carcinoma, dermatofibroma, vascular lesion and OOD lesions that do not fit into the other categories. Figure 2 illustrates an example of the training categories.

Together with the images, we provide information related to the anatomic location of the lesion, age, capture date, and sex of the patients. Our efforts were directed to create a diverse dataset with cases that dermatologist face in their usual clinical practice. We also present the results obtained after training six baseline algorithms to classify images into the eight lesion categories.

## Methods

### General

The BCN20000 dataset comprises 18946 high-quality dermoscopic images corresponding to 5583 skin lesions captured from 2010 to 2016. These images were collected by the Department of Dermatology at the “Hospital ClÃnic de Barcelona”, employing a set of dermoscopic attachments on three high-resolution cameras. Images were originally stored in a secure server using a directory structure organized by camera and capture month. Informed consent was obtained from all participants.

The dataset is published open source for research, to enable the training of classification algorithms such as artificial neural networks. The dataset is split into train and test sets, with 12413 and 6533 images, respectively. Diagnosis labels are only provided for the training set. The separation of the dataset into training and test sets is due to its inclusion in the broader ISIC database. Establishing a private test set is crucial for the challenges organized by the ISIC consortium^16,18,22^, allowing for unbiased evaluation of algorithmic performance. Specifically, all images classified as ‘unknown’ were assigned to the test set. For the remaining images, we employed stratified random sampling to create the training set.

### Image acquisition

**Fig. 3:**
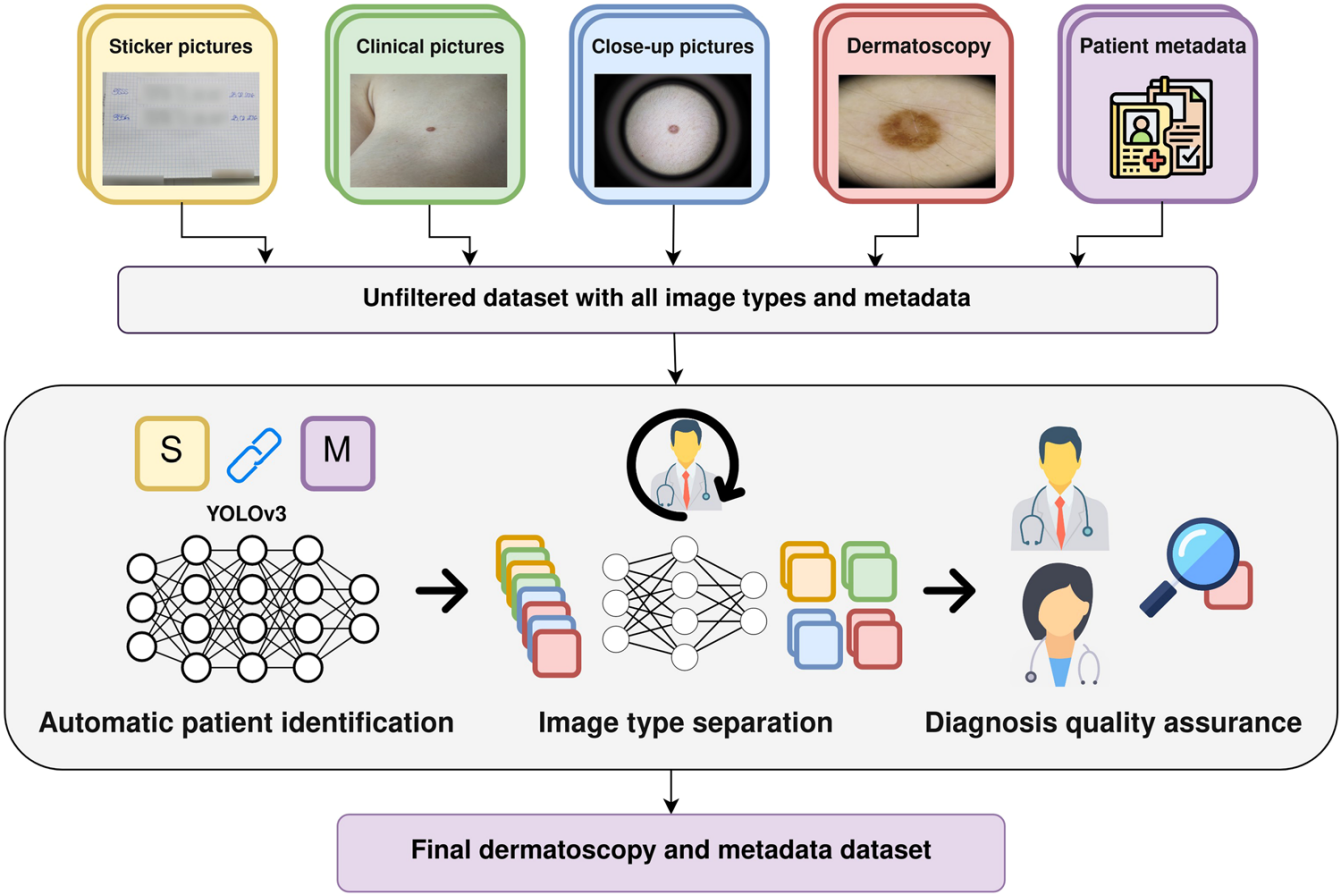
BCN20000 Dataset Preparation Pipeline. The process begins with the collection of images and metadata. A neural network is then employed to classify and separate between the image types. Patient identifiers are extracted from ‘Sticker pictures’ using a YOLOv3 network. Dermatoscopy’s diagnosis are revised by multiple reviewers for quality assurance. The resultant BCN20000 dataset is composed exclusively of dermoscopic images and metadata, divided into training and testing sets.

Every time a new patient came to the hospital, the doctors captured a photo of the identifier sticker containing the name and unique patient identifier. Then, the doctors captured several clinical and close-up pictures, and finally, they obtained one or more dermoscopic images, see Fig. 3. The total number of images captured from 2010 until 2016 is 94302. These images were organized in folders per camera and month of capture, without differentiation of the imaging types (sticker, clinical, close-up or dermatoscopy images). Images were collected and shared with institutional ethics approval number HCB/2019/0413.

### Image type separation

To segregate the four image types (sticker, clinical, close-up, and dermoscopic), we utilized a convolutional neural network based on the EfficientNetB0 architecture, incorporating a human-in-the-loop system for quality assurance. The usage of active learning minimized labelling effort. This classifier achieved a balanced accuracy of 98.9% on a validation set of 500 images, after being trained on 1,000 manually labelled images. The result was subsequently verified by expert dermatologists.

### Automatic patient identification

**Fig. 4:**
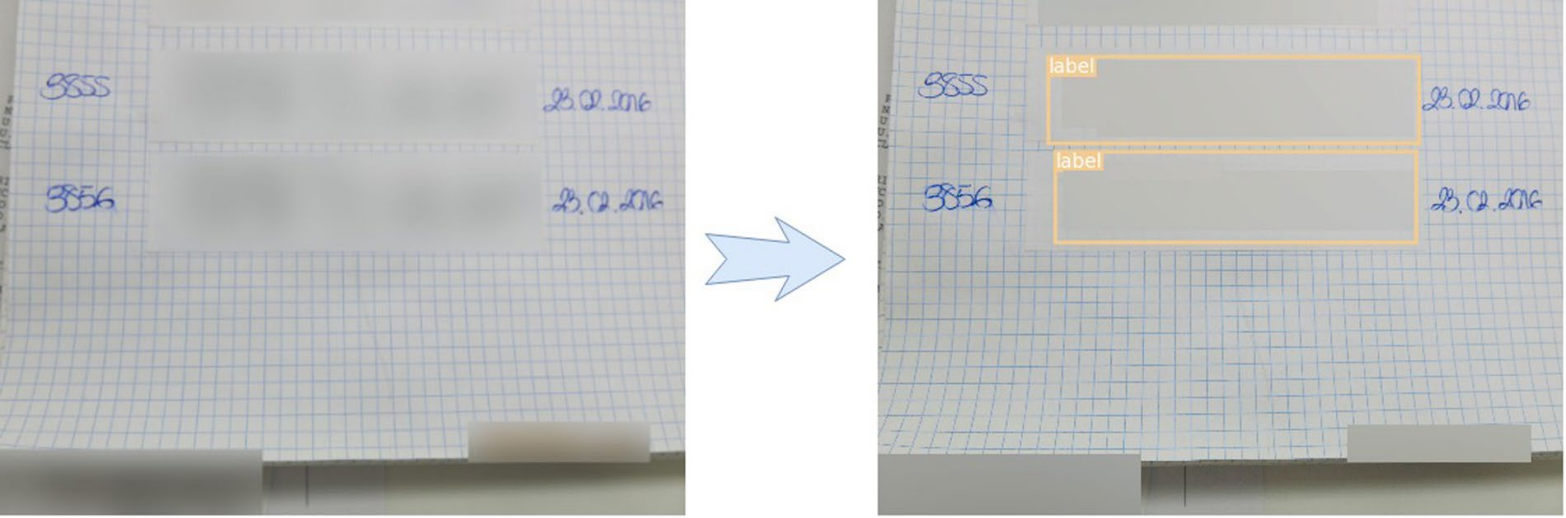
Example of the sticker detection algorithm. Left: original image (blurred for privacy), right: detection of the YOLOv3 Architecture.

The sticker images could contain the patient information at any location of the picture. To automate the information extraction, we trained a YOLOv3 architecture^23^ to determine the size and location of the sticker in the image. We trained the architecture on a subset of 200 hand-labeled images and validated it on 50 images obtaining an mAP of 0.72. This architecture was then used to detect the stickers in the rest of the images (see Fig. 4).

Following the automated detection of sticker locations, we used Tesseract OCR^24^ to read the patient name and identifier. Not all images presented the same kind of stickers. In addition, there was handwritten information on some sticker images, so the results were revised by image readers after the process was finished. Finally, after all the sticker images had been correctly identified, we propagated the label information to the consecutive clinical, close-up, and dermoscopic pictures.

### Linking images to patient reports

Patient names, identifiers, and image capture dates were used to assign one of the possible diagnostic categories, retrieved from several Excel files maintained by the doctors. Not all the images had a corresponding row in the diagnostic file since doctors would often take photos of lesions that were not clinically relevant and hence were of no interest for the construction of the dataset.

Finally, several readers revised each dermoscopic image to check the plausibility of the diagnostic category. Discrepancies were handled by multiple reader revisions and, if unanimous agreement amongst the reviewing panel was not achieved, they were discarded from the dataset. The metadata for each patient was gathered by the clinical practitioners at the time of capture. 

## Data Records

All data records of the BCN20000 dataset are released under a Creative Commons Attribution 4.0 International (CC-BY 4.0) license and are permanently accessible to the public through the Figshare repository with 10.6084/m9.figshare.24140028^25^. The dataset is partitioned into a training set comprising 12,413 images and a test set consisting of 6,533 images. Dermoscopic images and metadata are available for both sets. However, detailed diagnosis information is exclusively provided for the training set, encompassing eight distinct diagnostic categories.

Table 1 provides a comprehensive analysis of the training set, delineating the distribution of images stratified by diagnosis, sex, and anatomical location at both patient and lesion levels. The test set introduces an additional ‘unknown’ category, exclusive to it and absent from the training cohort. This category includes lesions that could not be definitively classified into the established categories, reflecting the complexity often encountered in clinical practice.

**Table 1:** The upper part of the table shows a summary of the Patient and lesion-level characteristics of the training set.

Training patient-level characteristics									
	NV	MEL	SCC	BKL	BCC	AK	DF	VASC	Total
Patient	4206 (34%)	2857 (23%)	431 (3%)	1138 (9%)	2809 (23%)	737 (6%)	124 (1%)	111 (1%)	12413 (100%)
Male	1946 (46%)	1465 (51%)	295 (68%)	625 (55%)	1683 (60%)	367 (50%)	59 (48%)	59 (53%)	6499 (52%)
Female	2219 (53%)	1379 (48%)	136 (32%)	502 (44%)	1120 (40%)	367 (50%)	65 (52%)	52 (47%)	5840 (47.5%)
Not-reported	41 (1%)	13 (1%)	0 (0%)	11 (1%)	6 (1%)	3 (1%)	0 (0%)	0 (0%)	74 (0.5%)
Age	43.4 ± 16	60.5 ± 16	72 ± 11	62.6 ± 15	65.5 ± 15	67.8 ± 13	50.4 ± 17	55.6 ± 17	56.8 ± 18
**Lesion location**									
Anterior torso	2346 (56%)	1127 (39%)	97 (23%)	292 (25%)	1049 (37%)	91 (12%)	39 (31%)	45 (40%)	5086 (41%)
Up. extremity	400 (9%)	380 (13%)	57 (13%)	78 (7%)	307 (14%)	62 (8%)	28 (23%)	7 (6%)	1319 (10%)
Lo. extremity	810 (19%)	489 (17%)	126 (29%)	206 (18%)	400 (11%)	57 (8%)	54 (44%)	31 (28%)	2173 (17%)
Head/neck	413 (10%)	635 (22%)	132 (31%)	523 (46%)	997 (36%)	500 (69%)	0 (0%)	23 (21%)	3233 (26%)
Palms/soles	156 (4%)	195 (7%)	13 (3%)	7 (1%)	0 (0%)	6 (1%)	0 (0%)	3 (3%)	380 (3%)
Oral/genital	24 (1%)	19 (1%)	0 (0%)	14 (1%)	0 (0%)	0 (0%)	0 (0%)	2 (2%)	59 (1%)
Not-reported	57 (1%)	12 (1%)	6 (1%)	18 (2%)	56 (2%)	21 (2%)	3 (2%)	0 (0%)	173 (2%)
**Test Unknown subclass distribution**									
	Nonprimary mel	Benign neoplasm	Scar	Infla-mmatory	Normal variant	Infectious disease	Other		
Count	632 (30.98%)	422 (20.69%)	395 (19.36%)	375 (18.38%)	116 (5.69%)	68 (3.33%)	32 (1.57%)		

The different classes are Nevus (NV), Melanoma (MEL), Squamous carcinoma (SCC), Basal cell carcinoma (BCC), Benign Keratosis (BKL), Dermatofibroma (DF), Actinic keratosis (AK) and Vascular (VASC). The lower section details the subclass distribution within the ‘Unknown’ category for the test set.

Significantly, the dataset includes images of lesions located in hard-to-diagnose areas, such as nails and mucosa. This aspect makes the BCN20000 dataset distinct from other more curated and publicly available datasets, as it more closely mirrors the challenges and diversity of actual clinical settings, which is lacking in current dermatoscopy datasets. The dermoscopy metadata encompasses patient age, biological sex, primary anatomic site of the lesion, date of capture, definitive lesion diagnosis, and designated data split.

### Dataset format

The dataset is composed of 18946 dermoscopic images and a “.csv” file with comma-separated values containing the image name, label information, and metadata for all patients. Images are encoded in Joint Photography Expert Group (JPEG) format^26^, and they are 1024 by 1024 pixels each.

### Dataset metadata

The dataset presents the following metadata:

- **BCN filename**: Unique identifier for each dermoscopy image file.
- **Age approximation**: Patient’s age quantized in 5-year intervals from 0 to 85.
- **Anatomical site of the legion**: Categorizes the lesion’s body location into six areas: anterior torso, upper extremities, lower extremities, head/neck, palms/soles, and oral/genitalia.
- **Diagnosis**: Specifies the lesion presented on the dermatoscopic image.
- **Lesion id**: A unique identifier for each lesion, noting that some lesions may be represented by multiple images.
- **Capture date**: The capture date of the photograph in YYYY-MM-DD format.
- **Sex**: The patient’s sex, recorded as male, female, other, or not-reported.
- **Split**: Designates the image’s allocation to either the test or training set.

## Technical Validation

### Histopathologic validation

Every melanoma diagnosis was confirmed through histopathologic examination of excised lesions by board-certified dermatopathologists, ensuring the highest level of diagnostic accuracy for melanoma classifications. Other malignant skin tumors also had histologic confirmation. Benign tumors were confirmed either histologically, through confocal examination, or by a clinical diagnosis from two experienced dermatologists at the referral center. Follow-up of tumors without excision confirmed stability to rule out malignancy.

### Additional diagnostic modalities

Some lesions were diagnosed utilizing reflectance confocal microscopy, which offers near-cellular-level resolution^27^, or by digital dermoscopic follow-up to confirm stability. These methods provide a non-invasive, yet reliable means of identifying non-melanoma skin conditions.

### Baseline CNN training

To assist researchers interested in automatic skin lesion classification of dermoscopy images, we developed and evaluated six baseline Convolutional Neural Networks (CNNs), which can serve as a starting point for future studies. The code can be found in the official repository at https://github.com/imatge-upc/BCN20000github.com/imatge-upc/BCN20000.

The implemented models are based on ResNet^28^ and EfficientNet^29^ architectures. We trained three small ResNet models with 18, 34, and 50 layers, respectively. From the EfficientNet architectures, we trained the B0, B1, and B2 models. We chose these architectures as they were used by the winners of the latest ISIC 2020 challenge^18^. All networks were pre-trained on ImageNet^30^. Our implementation uses the PyTorch framework for deep learning. We divided the data into training, validation, and testing with 75%, 5%, and 20% respectively.

During training, each RGB input image was resized to the appropriate model’s input size and augmented by performing the following random operations: resized crops with scales 0.8 to 1.2 and aspect ratio of 0.9 to 1.1, transforming from colour to grey-scale with 20% probability or applying permutation with 80% probability, including brightness (20%), saturation (20%), contrast (20%) and hue (20%), and horizontal and vertical flips (50%). Finally, the pixel values were normalized according to ImageNet mean and variance. In addition, we used weighted sampling to construct a uniform class distribution in the training batches to account for the severe class imbalance present in the dataset. We employed the Adam optimizer and used Cross-Entropy as the loss function throughout the training process. Finally, we trained the model for 130 epochs, or until the early stopping criterion was met, which involved halting training if the selected metric did not decrease for 20 consecutive epochs. The metric used to compare the models is balanced accuracy which measures the overall accuracy of a classification model by considering the proportion of true positive and true negative predictions for each class, making it desirable for unbalanced datasets. For the specific hyperparameters used during model training, refer to the official code repository https://github.com/imatge-upc/BCN20000.

**Fig. 5:**
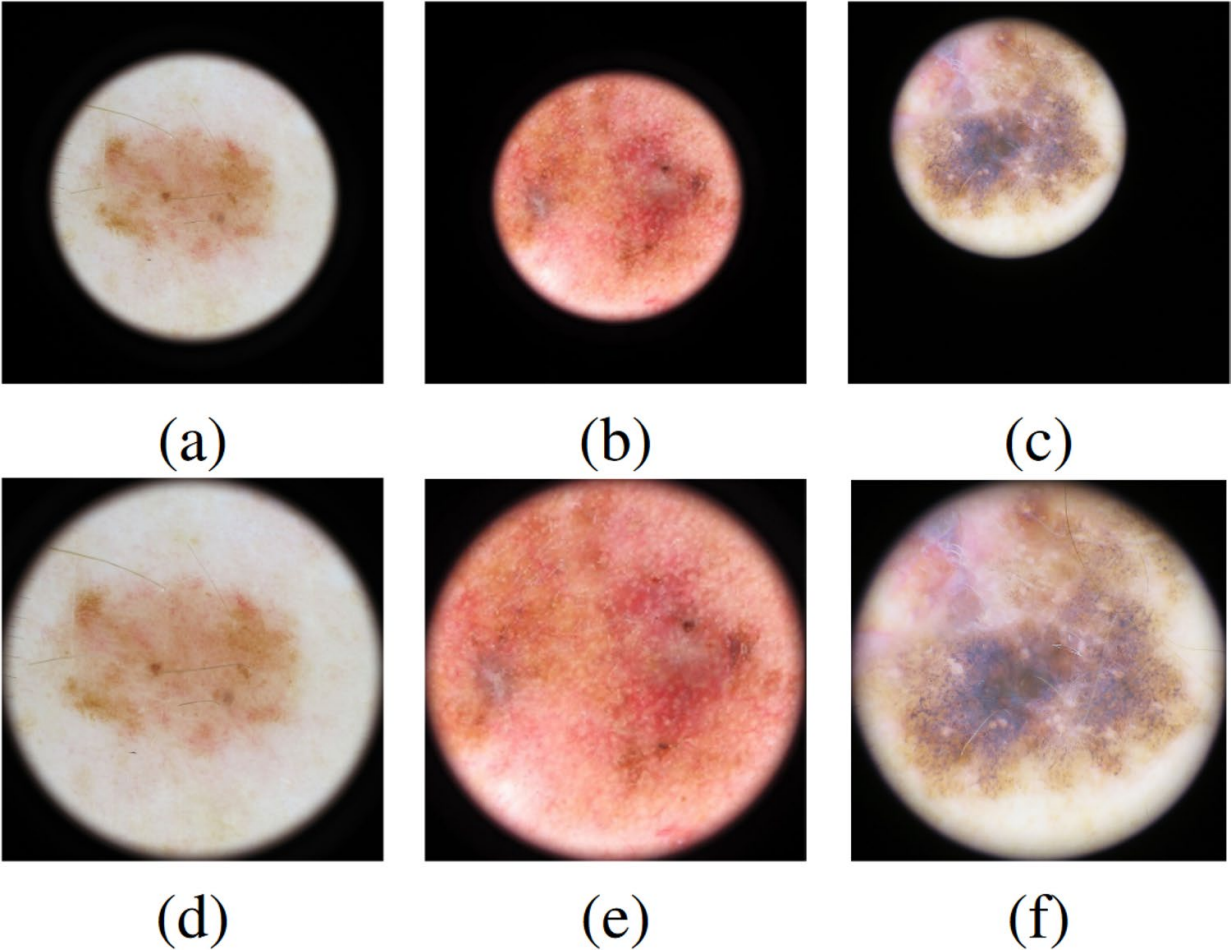
Comparison between original (**a,b,c**) and processed dermoscopic images (**d,e,f**) using the proposed image cropping algorithm.

Around 20% of the images in the dataset present a dark frame. This phenomenon would introduce biases in the models. We used a cropping algorithm to detect the Region Of Interest (ROI), and the lesion, and remove the dark background as much as possible. First, images were converted to grayscale and binarized with a low threshold, to retain the entire dermoscopy field of view. Then, we found the center of mass and the major and minor axis of an ellipse with the same second central moments as the ROI area. Next, based on these values, we derived a rectangular bounding box for cropping, covering the relevant field of view. Finally, we automatically determined the necessity of the cropping by comparing the mean intensity inside and outside the bounding box. Visual inspection showed that the method was robust. Examples of four different lesions before and after cropping are shown in Fig. 5. We fine-tuned the six pretrained architectures for the cropped and uncropped datasets and found a mean increase of 2.5% in balanced accuracy across all the architectures trained on the cropped dataset. Table 2 shows the balanced accuracy of each model for the cropped and uncropped datasets. The best results were obtained for the EfficientNet-B2 model on the cropped dataset, with a balanced accuracy of 0.461. The mean and variance were obtained using stratified k-fold cross-validation with five folds.

**Table 2:** Balanced accuracy achieved by different classifier architectures on the test set, both with and without image preprocessing.

Architecture	Uncropped	Cropped
ResNet18	0.4066 ± 0.017	0.4215 ± 0.020
ResNet34	0.4257 ± 0.019	0.4427 ± 0.047
ResNet50	0.4239 ± 0.025	0.4411 ± 0.020
EfficientNet-B0	0.4395 ± 0.025	0.4562 ± 0.045
EfficientNet-B1	0.4391 ± 0.011	0.4443 ± 0.022
EfficientNet-B2	0.4514 ± 0.025	**0.4605** ± **0.046**

## Usage Notes

The images in the BCN20000 dataset correspond to the following categories: nevus, melanoma, basal cell carcinoma, seborrheic keratosis, actinic keratosis, squamous cell carcinoma, dermatofibroma, vascular lesion and an extra OOD class only available on the test set. An example of each class on the training set can be found in Fig. 2. Each image is coupled with additional information regarding the anatomic location of the lesion, patient age and sex, image capture date, lesion ID, and diagnosis. The dataset was part of the ISIC 2019 and 2020 Challenge, where participants were asked to classify among various diagnostic categories and identify out of the distribution situations, where the algorithm is seeing a skin lesion it has not been trained to deal with.

### Description of diagnosis categories

- **Melanoma (MEL)**: Melanoma is a malignant neoplasm derived from melanocytes that may appear in different variants. Melanomas can be invasive or non-invasive (*in situ*). We included all common variants of melanoma, such as melanoma *in situ*, superficial spreading melanoma, nodular melanoma, lentigo malignant melanoma, acral lentiginous melanoma and mucosal melanoma.
- **Nevus (NV)**: Melanocytic nevi are benign neoplasms of melanocytes and appear in many variants. The variants may differ significantly from a dermatoscopic point of view. However, in contrast to melanoma, they are usually symmetric with regard to the distribution of colour and structure in typical nevi and more irregular in atypical nevi.
- **Basal cell carcinoma (BCC)**: Basal cell carcinoma is a common variant of epithelial skin cancer that rarely metastasizes but grows destructively if untreated. It appears in different morphologic variants (flat, nodular, pigmented, cystic, infiltrative, morpheiform, etc).
- **Squamous carcinoma (SCC)**: It develops in the squamous cells that make up the middle and outer layers of the skin. Squamous cell carcinoma of the skin is usually not life-threatening, though it can be aggressive. Untreated, squamous cell carcinoma of the skin can grow large or spread to other body parts, causing severe complications.
- **Dermatofibroma (DF)**: Dermatofibroma is a benign skin lesion regarded as either a benign proliferation or an inflammatory reaction to minimal trauma. The most common dermatoscopic visual clue are reticular lines at the periphery with a central white patch denoting fibrosis.
- **Benign Keratosis (BKL)**: “Benign keratosis” is a generic class that includes seborrheic keratoses (“senile wart”), solar lentigo - which can be regarded as a flat variant of seborrheic keratosis - and lichen-planus like keratoses, which corresponds to a seborrheic keratosis or a solar lentigo with inflammation and regression. The three subgroups may look different dermatoscopically, but we grouped them because they are similar biologically and often reported histopathologically under the same generic term. From a dermatoscopic view, lichen planus-like keratoses are especially challenging because they can show morphologic features mimicking melanoma and are often biopsied or excised for diagnostic reasons. In addition, the dermatoscopic appearance of seborrheic keratoses varies according to anatomic site and type.
- **Actinic keratosis (AK)**: is a rough, scaly patch on the skin that develops from years of sun exposure. It’s often found on sun-exposed areas such as the face, lips, ears, forearms, scalp, neck, or back of the hands. Left untreated, the risk of actinic keratoses turning into a type of skin cancer called squamous cell carcinoma is about 5% to 10%.
- **Vascular (VASC)**: cutaneous vascular lesions are benign lesions which may mimic malignant skin tumours. Several vascular diseases are included in this class, such as capillary angioma, hemangioma, pyogenic granuloma, cavernous angioma, venous angioma, verrucous angioma, lobed angioma, dermal angioma, thrombosed angioma, venous lake, haemorrhoid, ectatic vascular structure, or thrombosed vascular structure.

## Data Availability

The code used for the pre-processing of the images and the training of the different architectures seen in Fig. 5 can be found at https://github.com/imatge-upc/BCN20000. All code is licensed under the Attribution 4.0 International (CC BY 4.0) License.
